# Awareness and Self Care Practices of Elderly Men Regarding Prostate Diseases in Karachi, Pakistan

**DOI:** 10.7759/cureus.4617

**Published:** 2019-05-08

**Authors:** Mahpara Tanveer, Faryal Tahir, Zainab Majid, Muhammad Mustafa Hussain, Sarrah Ali Asghar, Laila Tul Qadar, Jabran Wasti, Ashar Shahid, Iqra Tasleem, Hamza Aijaz Khan, Mehroz Sami, Qazi Arsalan

**Affiliations:** 1 Internal Medicine, Dow University of Health Sciences, Karachi, PAK

**Keywords:** prostate, prostate cancer, adult men, screening, awareness, pakistan

## Abstract

Background: The prostate gland is a male sexual organ which has a number of diseases associated with it, such as prostatitis, benign prostatic hyperplasia (BPH), and prostatic cancer (PC). BPH is the most common neoplasm, and it causes significant urinary symptoms in adult males. According to the World Health Organization (WHO) report of 2014, out of all the cancers, PC had the fifth highest incidence in males throughout Pakistan. The purpose of this study was to determine the awareness of elderly men of Karachi regarding prostate diseases (PDs) and their attitude towards screening practices.

Method: From September to December 2018, a cross-sectional study was performed among 450 men of Karachi older than 50 years of age. Frequencies and percentages were evaluated for categorical variables using Statistical Package for Social Sciences (SPSS), version 22 (IBM SPSS Statistics, Armonk, NY). Chi-square test was used to determine if there was any significant difference. A p-value of < 0.05 was considered significant.

Result: A total of 350 respondents answered the questionnaire. The mean age of the respondents was 61 years. Almost half of the population had heard about the prostate but 64% did not know any PDs, whereas 48% were aware of the increased risk of PDs in the elderly. Almost 48% of the respondents believed that age was the main cause of disorders involving the prostate. Even though the knowledge was lacking, people had an overall positive attitude. Most respondents (66%) had the attitude that all adult men must undergo prostate screening. The majority of respondents (85%) had not undergone screening of the prostate as the huge group of participants (86%) had not been advised to do it. Upon statistical testing, having heard about the prostate and undergoing prostate screening in the past (p = 0.008 and 0.024, respectively) was significantly associated with age. Having prior experience with prostate screening was also associated significantly with marital status (p < 0.001).

Conclusion: Respondents have inadequate knowledge about PC but a good attitude about undergoing prostate examination. It is absolutely crucial to increase information on the risks of PDs, particularly PC, and the benefits of early detection.

## Introduction

The prostate, a walnut-sized gland, is an integral part of the male reproductive system that wraps around the urethra between the pubic bone and the rectum, below the bladder. With age, its size increases and can be much more sizeable in older men. Apart from age, the main causes of prostatic hyperplasia (PH) include family history, ethnic background, excessive dihydrotestosterone (DHT) levels, obesity, diabetes, sedentary lifestyle, and poor diet [1]. An enlarged prostate can block urine flow through the urethra, consequently causing frequent urination, urinary tract infections (UTIs), hematuria, and urinary incontinence [2]. Apart from hyperplasia, prostate cancer (PC) and prostatitis are also the major concerns in elderly men. Globally, the second most commonly diagnosed cancer is PC, affecting 15% of men and nowadays believed as one of the leading causes of mortality due to cancer among men [3].

In the social learning theory, Bandura postulated that knowledge regarding PC raises awareness about the risk factors and propensity towards positive health-seeking behavior which includes prompt screening, diagnosis, treatment, and adherence to the respective treatment [4]. Knowledge can be attained on the individual level from influential models, such as elder siblings, friends, parents, teachers, and healthcare providers. Literature, media, movies, and internet also play a very essential role in the delivery of knowledge and awareness [4]. A study conducted on the behalf of YouGov for "Prostate Cancer UK" has highlighted that only two in five people (40%) know that the age of 50 years or over can lead to an increased risk of PC in men [5]. Digital rectal exam (DRE) and prostate-specific antigen (PSA) are the fundamental investigations performed for clinical evaluation of prostate gland.

Attitude and beliefs may be as crucial as knowledge of screening behavior. A current study conducted in the UK reported that although both white and black men had relatively higher knowledge about the major risk factors linked with PC, 20% of the white men (whereas only 5% of the black men) had been evaluated for PC [6]. The same study highlighted that black men felt more embarrassed by their symptoms of PC than white men and were less comfortable in consulting their physician about them. According to a population-based study conducted in Southwest Nigeria, 42.6% of the participants had a good response towards screening and treatment of prostatic diseases (PDs), whereas the remaining 57.4% of the participants showed a poor attitude [7].

Although PDs have received much media attention, studies on the public’s knowledge, attitudes, and beliefs concerning the prostate are scarce in our region. Therefore, the primary objective of the current study was to evaluate the knowledge, attitude, and self-care practices of aged men regarding diseases primarily affecting the prostate. Such an attitude should rely on a firm background of genuine information and motivation from physicians regarding prostate screening. The point of emphasis is that the lack of knowledge not only impedes the awareness for the screening program but also has a negative impact on men's health preferences at the individual level. Thus, the secondary objective of this study is to empower the masses with the basic essence of prostate and prostate-related knowledge, rectify unwilling attitudes towards the dilemma, and recommend to the population useful and timely prostate practices. In this way, the clinical conditions can be treated and managed properly by medical personnel and further policies can be crafted by the researchers.

## Materials and methods

A multicenter cross-sectional study was conducted between September to December 2018. Based on the assumption that 50% of the general population had adequate knowledge regarding PDs, the sample size (using 95% confidence interval and at a degree of precision of 5%) was determined to be 384 subjects. This was increased to 455 to attain maximum representation. Eligible participants were selected on the basis of non-probability convenience sampling. Participation in the study was voluntary, and the aim of the study and its impact was explained to each participant by the investigator in the written informed consent form before they were asked to fill out the questionnaire. Since the risk for PDs increases significantly after the age of 50 in men, our target population was elderly men aged between 50 and 79, men who could communicate easily, and those who had no psychiatric disorder. In order to minimize bias from professional knowledge, healthcare professionals were not included in the study. Men suffering from any of the PDs were also excluded.

Survey design

A self-administered 18 question research form, designed after going through several questionnaires from similar published studies, was formulated as a tool for data collection. The questionnaire was translated into the Urdu language to allow the natives to complete it conveniently. It was back-translated to English to ensure its validity.

The questionnaire was divided into four sections. The first section consisted of demographics, including age, marital status, family history, education, occupation, and residence. The remaining three parts assessed the participant’s knowledge, attitude, and practice via a series of close-ended questions. The knowledge section consisted of awareness regarding the prostate, its disorders, and its seriousness. The perception of people towards the prostate examination, its perceived benefits, and towards the early detection of PC was included in the attitude section. Lastly, practices towards screening tests were assessed in the fourth section. The proforma was verified by a urologist, pathologist, and oncologist. In order to pretest the form, a pilot study was conducted on 45 participants, following which necessary changes were made in the questionnaire and those responses were excluded from the final results. Partially completed forms were excluded from the study and no imputation methods were used. The population-based sample was selected from different areas of Karachi using convenience sampling. Before administering the questionnaire, the objective and benefits of the study were explained to the participants and their verbal consent was obtained. Out of the 455 eligible men, five men refused to participate; hence, the cooperation rate was recorded as 98.9%.

Statistical analysis

Data were analyzed using the Statistical Package for Social Sciences (SPSS), version 22 (IBM SPSS Statistics, Armonk, NY). Frequencies and percentages were evaluated for categorical variables, whereas for continuous variables, the mean and standard deviation were reported. The Chi-square test was used to determine if there was any significant difference. A p < 0.05 was considered as being significant.

## Results

Of the target population, almost half of the respondents were between the ages of 45 to 60, while the remaining were elderly. The mean age of the respondents was 61 years. Most of the respondents were married (83.1%). The majority of the respondents had completed their graduation (35%), whereas 19.6% of the population was uneducated. These demographics are displayed in Table 1.

**Table 1:** Demographics of the Respondents

Characteristics	Frequency	Percentage (%)
Age		
45 - 60	229	50.9
> 60	221	49.1
Education		
Uneducated	88	19.6
Primary	78	17.3
Secondary	126	28.0
Graduate and above	158	35.1
Marital Status		
Married	374	83.1
Divorced	20	4.4
Widowed	28	6.2
Never Married	28	6.2

**Figure 1:**
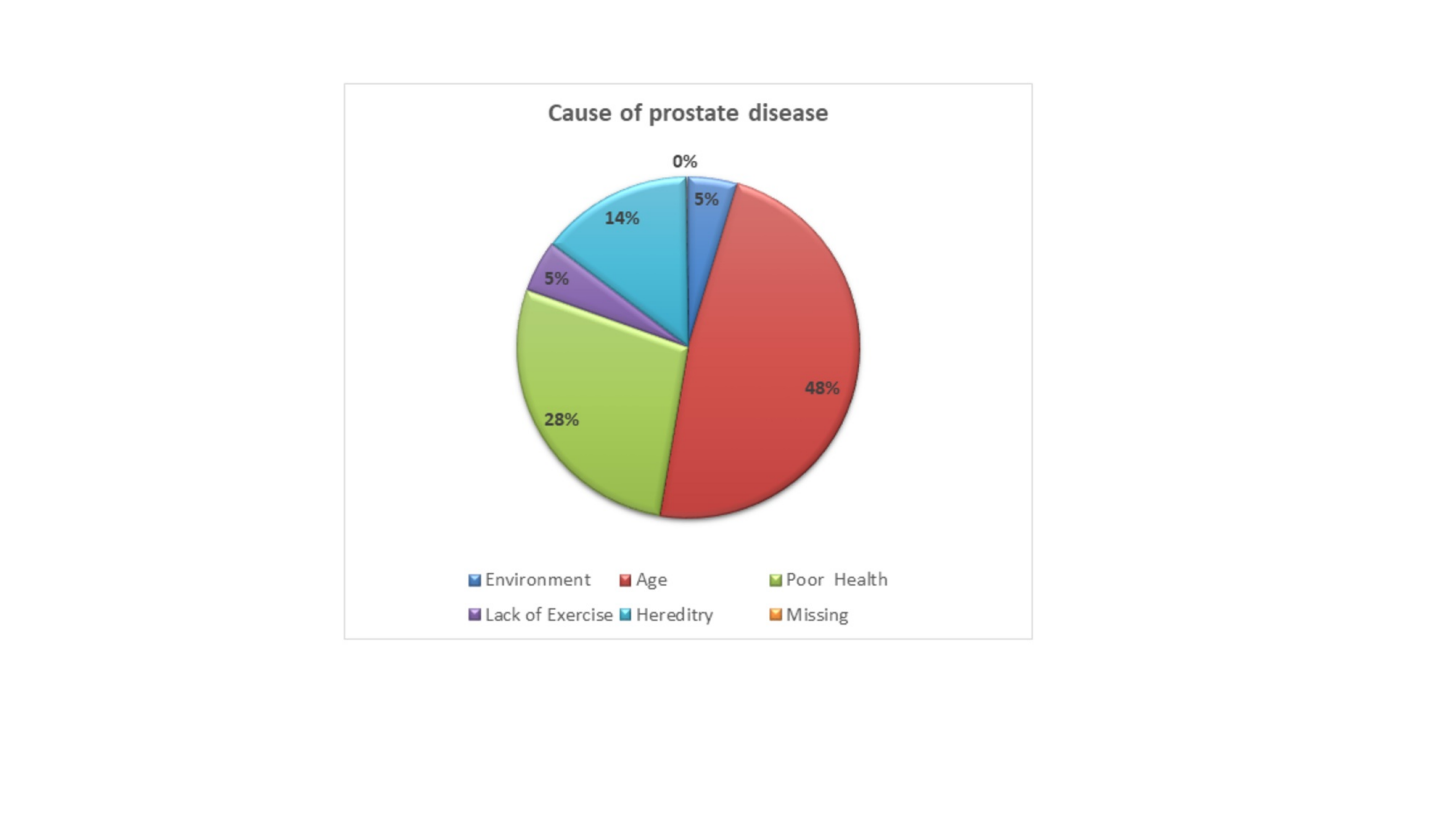
Causes of Prostate Diseases

**Figure 2:**
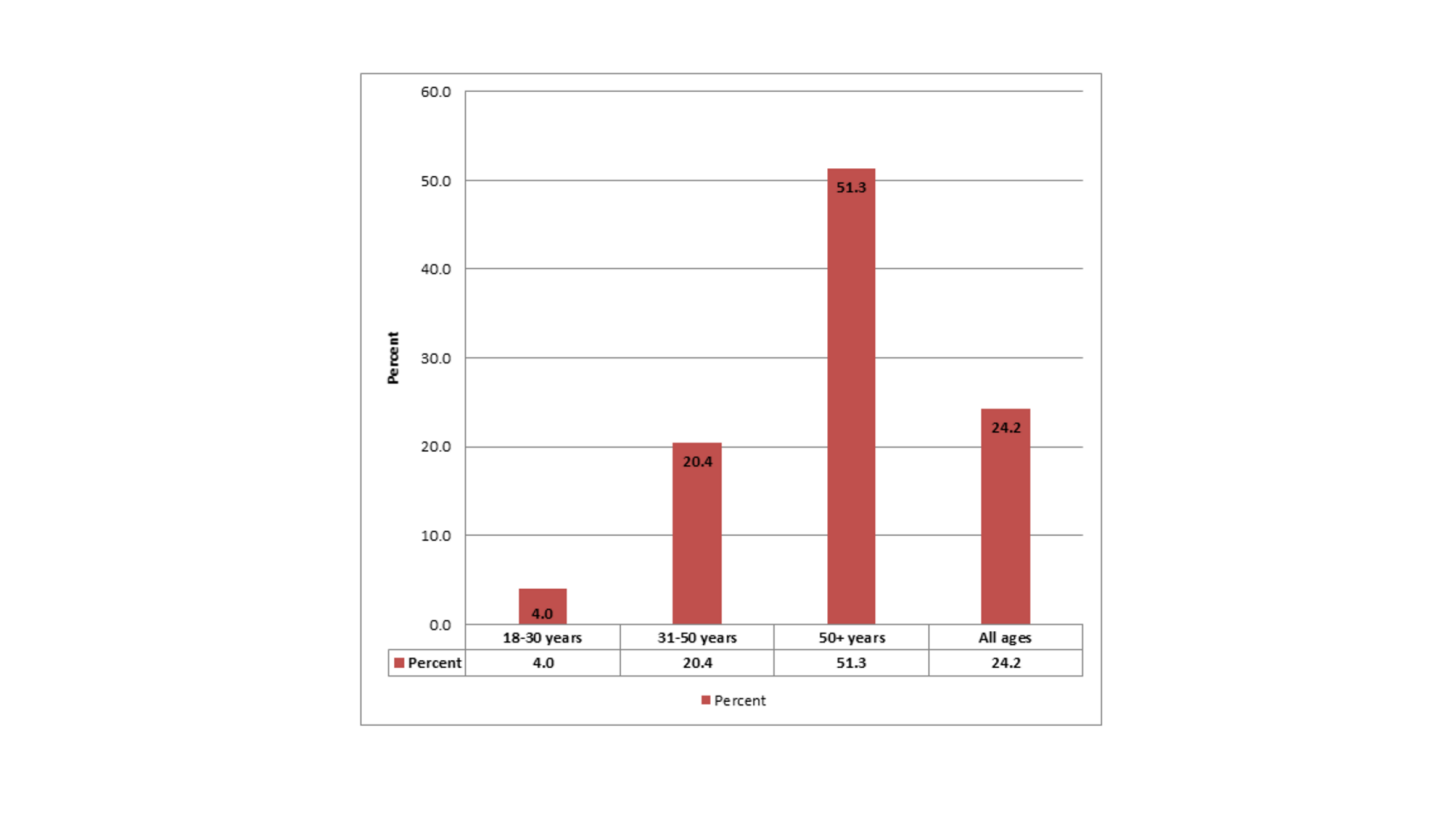
Prevalence of Prostate Diseases with Respect to Age Groups

When tested on their knowledge as shown in Table 2, almost half of the population had heard of the prostate; however, the majority (64%) of the people were not aware of any PDs. Nonetheless, about half (48%) of the population was aware of the increasing risk of PDs in the elderly. When asked about the probable cause of such diseases, the same proportion (48%) believed that age is the major factor, whereas the environment was thought to play the least significant role here (Figure 1). Over half of the people correctly knew that PDs are more prevalent in people above 50 years of age (Figure 2). The practices of our participants regarding prostate screening are summarized in Table 3.

**Table 2:** Respondents' Knowledge of the Prostate PC: prostate cancer; PDs: prostate diseases

Knowledge	Yes (%)
Heard of the prostate before	229 (50.9)
Knowledge of any disorder of the prostate	161 (35.8)
Does the incidence of PDs increase with age?	211 (46.9)
Weak urine is a symptom of PDs	160 (35.6)
A man is more likely to develop PC if his father had it	105 (23.3)

**Table 3:** Prostate Screening Practice

Practices	Frequency (%)
Advised to go for prostate screening before	68 (15.1)
Underwent prostate screening before	61 (13.6)

The majority of the participants (85%) were not screened for PDs. 'Not being advised by the doctor' (33%) was the most common reason for not seeking medical screening, followed by the absence of symptoms (23%). However, most of the people (64%) chose to visit a doctor when there was the appearance of any symptoms. Pursuing homeopathic treatment was the second most popular answer as shown in Tables 4-5.

**Table 4:** Responses for Never Having Prostate Screening Before (n = 389)

Reasons for not having prostate screening before	Frequency (%)
No time	38 (9.77)
Not advised	131 (33.7)
High cost	33 (8.48)
No need	73 (18.7)
Lack of awareness	13 (3.34)
No symptoms	90 (23.1)
Missing values	11 (2.83)

**Table 5:** Responses for Treatment Choice If Participants Experience Frequent and Painful Urination

Treatment choice in case of frequent and painful urination	Frequency (%)
Home remedy	43 (9.6)
Improve diet	48 (10.7)
Homeopathy	72 (16.0)
Visit a doctor	287 (63.8)

Even though the knowledge was lacking, people had an overall positive attitude. Most participants (66%) had the attitude that all adult men must undergo prostate screening. Beliefs like early detection of PC can lead to complete cure (53%) and medical and surgical treatment can cure PC (53%) were quite common. Table 6 shows the attitude of men towards PDs.

**Table 6:** Responses on Participants' Attitude About Prostate Diseases DRE: digital rectal exam; PC: prostate cancer

Attitude	Yes (%)	No (%)
All adult men should go for prostate screening	297 (66.0)	34 (7.6)
Do you think early detection of PC can decrease complications?	280 (62.2)	44 (9.8)
Would you feel nervous or embarrassed if the doctor asked for DRE (examination by inserting a finger through the rectum)?	124 (27.6)	180 (40.0)
Medical and surgical treatment can cure prostatic problems	240 (53.3)	36 (8.0)
If you don’t have any symptoms, you don’t have PC	187 (41.6)	63 (14.0)

Upon statistical testing, having heard about prostate disorders and undergoing prostate screening in the past was significantly associated with age (p = 0.008 and 0.024, respectively). Having at least a secondary education also had a significant relationship with the former (p < 0.001). Having prior experience with prostate screening was also associated significantly with marital status (p < 0.001).

## Discussion

Knowledge among the masses about PDs is crucial for their early detection and diagnosis. However, we found that the awareness regarding the prostate and its diseases in elderly men to be extremely low. Previous studies also suggest the same lack of knowledge in terms of risk factors and symptoms of PDs. In a study by Steele et al., many men (50 years and older) residing in the state of New York were misinformed about their risk of PC [8]. Although our data clearly shows that age is believed to be the single most important risk factor, it is one of the few well-established risk factors for PDs (black race and family history being others) [9]. Moreover, Zainal et al. [10] also reported that out of the 200 respondents interviewed in their study, only 20 of them (10.0%) were aware of prostatic symptoms. This is on par with our data showing that a clear majority was not aware of weak urine being a symptom of PDs. These findings may be attributable to the association between the level of knowledge and education status among the respondents [7]. Our data also shows that the majority of the respondents never had a screening test (PSA or DRE) with ‘not being advised’ as the most common reason, followed by ‘lack of symptoms’. Previous studies also found the same reasons among men not undergoing screening [11-14]. According to a cross-sectional study by Morlando et al. [14], among those that had undergone the PSA test, 52.9% were recommended by a physician. In another study engaging black American men [15], a positive relationship between a doctor’s encouragement to be screened and patients having both PSA and DRE was established. In Pakistan, most of the people do not have health insurance; therefore, it could be understood that a routine check-up is rarely undertaken and thus results in the lack of the usual counseling about screening. As such, doctors, especially general physicians (GPs), are expected to play a significant role in any future policies regarding PD screening practices. Overall, there was a positive attitude towards PDs. With most of the respondents having never been screened or advised to undergo screening before, it was uplifting to find that most of them believed that all adult men should go for screening and that early detection would lead to better outcomes. This may be due to men’s knowledge of age being a risk factor and their belief in a possibly efficacious treatment as explained by the health belief model [16]. Furthermore, our data show that most men would not feel nervous or embarrassed if the doctor asked for a DRE. However, whether it is only true for symptomatic patients or as a screening tool in asymptomatic but high-risk patients remains to be elucidated.

We also found that most of the men were not sure that positive family history was a risk factor. This may be due to a lack of knowledge about diseases, specifically cancers, having a genetic predisposition. Likewise, Bloom et al. [17] reported that men with a family history did not perceive their risk to be higher than those without a family history. This is particularly alarming, as in a systematic review conducted by Johns and Houlston [18], it was established that men with a family history of PC had a significantly greater risk of developing the disease and thus screening these men might prove cost-effective. In a developing country like Pakistan, financial constraints remain a massive obstacle in preventing and curbing diseases. Should a strategy be developed for the early detection of PDs, it is evident that high-risk men would be preferably screened. This, in turn, depends on men having information at least about the major risk factors, of which family history is unarguably an important one to be aware of. In our study, we also found that out of 158 men who had completed graduation, 122 had heard about the prostate, 84 at least knew of some disorder of the prostate, 93 identified age as a risk factor, and 82 thought that a man is more likely to have PC if his father had it. This amounts to better knowledge than any other educational group. Moyo et al. [19] also reported that awareness of PC is more common among men with tertiary education. The literature shows that men with good knowledge are more likely to adopt screening practices [20-22]. As such, the importance of educational intervention to increase awareness regarding PDs and screening practices is understandable. Awareness campaigns and educational programs using videotapes and print-based materials can be used for dissemination of information. In this regard, videotapes have been shown to significantly increase knowledge in experimental groups [23]. Moreover, the literature shows that interventions based on the health belief model (HBM) also proved successful in increasing men’s knowledge. According to the HBM, personal beliefs about a disease determine the individual’s health behavior [24]. In a study by Zare et al. to assess the effect of HBM-based education on knowledge and PC screening behaviors [25], the mean scores of the perceived susceptibility, severity, barriers, and benefits increased significantly after the intervention. Therefore, it is important that surveys or other strategies to determine perceptions of men regarding PDs be developed, leading to HBM-based educational programs imparting knowledge among men.

Furthermore, the physician's role remains an integral part of improving the patient's knowledge and screening practices. Our data shows that the majority of the respondents believed that they would visit a doctor if they experienced urinary symptoms. In such circumstances, a discussion with the doctor regarding PC and counseling about tests for diagnosis might improve the patient's knowledge. In the era of shared decision-making (SDM), a process involving clinician-patient discussion of the pros and cons of screening has shown that men who had undergone a PSA test were more likely to report engaging in SDM [26]. As such, clinicians must engage their patients in a conversation, as permitted by time, to inculcate among them a better knowledge about PDs, leading to increased screening practices.

There are several limitations in our study that need to be considered. Firstly, in the minority of the respondents who did have some knowledge about the prostate, their source of information was not identified. Secondly, knowing that it was medical students conducting the study, who also translated into Urdu the components of the questionnaire for the participants, the respondents may not have given honest answers to questions for fear of being labeled as ignorant. Thirdly, misconceptions regarding PDs were not assessed.

## Conclusions

It can be concluded that there is inadequate knowledge about the prostate and its diseases among the elderly men of Karachi. Considering the prevalence of PC and benign prostatic hyperplasia (BPH), this is alarming. However, a positive attitude regarding the idea of regular screening practices was developed. Early recognition of the symptoms of PDs is vital for a timely diagnosis, thus reducing morbidity and mortality. We believe that awareness campaigns to publicize the risk factors and easy prevention of PDs will be helpful in this regard. Also, the counseling of patients by physicians will play a major role in curbing the problem.
